# Popularity and impact of using smart devices in medicine: experiences in Saudi Arabia

**DOI:** 10.1186/s12889-018-5465-y

**Published:** 2018-04-20

**Authors:** Sameer Al-Ghamdi

**Affiliations:** Department of Family Medicine, College of Medicine, Prince Sattam bin Abdulaziz University, Al Kharj, Saudi Arabia

**Keywords:** Smartphone, Smart device, Medicine, Saudi Arabia

## Abstract

**Background:**

The present study aimed to investigate smart device medical apps currently preferred by physicians in Saudi Arabia and the perceived impact of the apps on patient care.

**Methods:**

Questionnaires for this cross-sectional study on smart device medical apps were randomly emailed to 384 physicians registered in the Saudi Commission of Health Specialists database. A total of 300 physicians returned completed questionnaires, with a response rate of 78.5%. Physician demographics and their perceptions of medical apps were assessed, including questions on the purpose, impact, and types of medical apps used. Questions were answered using a Likert scale (1 = strongly disagree, 2 = disagree, 3 = not sure, 4 = agree, and 5 = strongly agree).

**Results:**

Study subjects had a median age of 39 years (57.7% male). Most respondents (88.3%) had smart devices, and 86.3% had at least one medical app installed. Just over half used an app at least once a day (53.0%). Medical apps were positively perceived, with physicians reporting increased dependency on the apps (Likert score: 4.7 ± 0.5).

**Conclusion:**

Medical apps were perceived to positively impact education, physician efficiency, and patient care.

**Electronic supplementary material:**

The online version of this article (10.1186/s12889-018-5465-y) contains supplementary material, which is available to authorized users.

## Background

The use of smart device applications (apps) in the medical setting has been steadily increasing in recent years [1, 2]. This is generally beneficial because smart device medical app use has been shown to increase physician productivity [1–4], efficiency [3, 5–7], and accuracy [3, 6, 8, 9] and to improve patient access to medical care [3, 10]. However, medical apps have received both positive and negative reviews from practicing physicians [10]. Therefore, identifying the best app was important. Medscape, UpToDate, Lexicomp, and Epocrates have received positive ratings and are most commonly used by healthcare workers [11]. In contrast, other applications were poorly rated because of low performance that did not meet healthcare workers’ needs. Some issues raised include app reliability [12, 13] and diagnostic accuracy [12–17], along with patient health disparities and poor health knowledge [18].

Smart phone medical apps are considered a major improvement to medicine, particularly in heavily populated countries [1, 3], which can be socioeconomically diverse. Saudi Arabia’s population is also socioeconomically diverse and many people cannot afford medical insurance or to pay for their medical care directly. Because medical apps improve physician efficiency [3, 5, 6] and productivity [1–4], they may help reduce hospital congestion, improve access to medical care, and reduce medical care costs. Therefore, it is important to understand how medical apps can improve patient healthcare and which apps will be most useful to specific healthcare systems. Here, we examine current physician use of smart device medical apps in Saudi Arabia and their perceived impact on patient care.

## Methods

### Sample and procedures

All data were collected using an anonymous questionnaire based on a previously published study [19]. Names of potential study participants were obtained from the Saudi Commission of Health Specialties database. Physicians were randomly selected using a simple random sampling method implemented with a computer random number generator. Invitations to participate in the study were sent by email to the addresses in the database.

In order to ensure that the sample was highly representative of the study population, the sample size was calculated according to the following formula (OpenEpi 2017): *n* = [DEFF*Np(1-p)]/[(d^2^/Z^2^_1-α/2_*(N-1) + p*(1-p)], where n = the calculated sample size, N = the total study population size (86,756 physicians [20]), DEFF = design effect (for cluster surveys, 1%), p = hypothesized %frequency of the outcome factor in the population (considered to be 50%), and d = the confidence limits as ±percent of 100 (5%). According to this formula, the sample size was calculated to be 382. Invitations to participate in the study were sent to 384 medical practitioners. A total of 300 physicians returned completed questionnaires, with a response rate of 78.5%.

The study questionnaire consisted of several parts. Part 1 collected participants’ demographic data, including age, gender, whether or not a mobile device was used, whether or not medical apps were installed on a smart device, the purpose of installed medical apps, and whether or not their hospital/institute recommended specific medical apps. Part 2 assessed the participants’ perception of smart device medical apps. Part 3 assessed the impact of medical apps on clinical training and practice. Part 4 evaluated what medical apps were used. Responses to questions in parts 2–4 were based on a 5-point Likert scale: 1 = strongly disagree, 2 = disagree, 3 = not sure, 4 = agree, and 5 = strongly agree. Questionnaires that were only partially completed were not included in the analysis (Additional file 1).

### Inclusion criteria

Being a licensed physician registered in the Saudi Commission of Health Specialties database and willing to participate in the study were the inclusion criteria of the study, and the questionnaire was in English.

### Statistical analysis

Data were analyzed using SPSS statistical software (version 21, SPSS, Inc., Chicago, IL, USA). Subject characteristics are presented as frequencies (percentage). Quantitative Likert scale data are presented as mean ± standard deviation.

### Ethical considerations

This prospective, cross-sectional study was reviewed and approved by the Deanship of Scientific Research at Prince Sattam bin Abdulaziz University (No. 2017/03/3361). The study did not involve any medical examination, surgical procedure, or collection of personal health information. Therefore, completing and returning the questionnaire was considered as provision of informed consent to participate in the study. The study was conducted in adherence to the tenets of the Declaration of Helsinki.

## Results

### Demographic characteristics of and smart device use by physicians in Saudi Arabia

Study subjects had a median age of 39 years (range: 26–67 years), and 57.7% subjects were male. Additionally, 43.0% of subjects were resident medical physicians, 33% were registrars, 12.3% were senior registrars, and 11.7% were consultants.

Approximately 88.3% of subjects used smart devices. All participants were aware that medical apps are available for smart devices and 86.3% had medical apps installed on their smart devices. Additionally, 88% of subjects reported that their hospital/institute recommended a specific medical app for their device. Participants reported using installed medical apps at least once a day (53.0%), at least once a week (35.3%), or less than once per month (11.7%, Table 1).

**Table 1:** Demographic characteristics of and smart device use by physicians in Saudi Arabia (*n* = 300 subjects)

		Number	Percent
Gender	Male	173	57.7
	Female	127	42.3
Medical rank	Resident	129	43.0
	Registrar	99	33.0
	Senior registrar	37	12.3
	Consultant	35	11.7
Are you using smart devices?	No	35	11.7
	Yes	265	88.3
Are you aware of the availability of medical apps on smart devices?	Yes	300	100.0
Have you installed medical apps on your smart device?	No	41	13.7
	Yes	259	86.3
Has your hospital/institution ever recommended that you obtain a specific medical app for your mobile phone?	No	36	12.0
	Yes	264	88.0
Frequency of use of installed medical apps	≥once/day	159	53.0
	≥once/week	106	35.3
	≤once/month	35	11.7

### Purpose of using installed smart device medical apps and frequency of use of installed medical apps

Installed smart phone medical apps were most commonly used for revision of medical knowledge (82.3%), for presentation preparation (75.3%), during ward rounds (71%), for medical information look-up (70%), for medical news updates (70%), for clinical skills guidance (69.7%), and for medical journal viewing (68.7%). Other usages included viewing of medication and drug guides (65%), preparing for exams (65.7%), and viewing of general clinical textbooks (53.3%). Approximately 12% of medical practitioners reported that they did not have a medical app installed (Table 2).

**Table 2:** Purpose for using installed smart device medical apps (*n* = 300 physicians)

	Number	Percent
Reviewing medical knowledge	247	82.3
Preparing presentations	226	75.3
During ward rounds	213	71.0
Reading medical news	210	70.0
Looking up medical information	210	70.0
Clinical skills guide	209	69.7
Reading medical journals	206	68.7
Exam preparation	197	65.7
Medication or drug guide	195	65.0
General clinical textbook	160	53.3
I do not have medical apps	36	12.0

Female medical practitioners at junior, registrar, or below ranks used installed medical apps at least daily or once a week compared with their counterparts with chi-square = 47.265, *p* < 0.001; age with the chi-square = 318.273, *p* < 0.001; and medical rank with the chi-square = 374.4, *p* < 0.001, respectively, as shown in Table 3.

**Table 3:** Gender and medical rank with the frequency of use of installed medical apps

		Frequency of use of installed medical apps			Total	Chi-square test	df	*p*-value
		At least once a day	At least once a week	Less than once a month				
Gender	Male	66	72	35	173	47.265	2	.000
	Female	93	34	0	127			
Medical rank	Resident	94	35	0	129	374.400	6	.000
	Registrar	65	34	0	99			
	Senior registrar	0	37	0	37			
	Consultant	0	0	35	35			
Age	< 30 years	51	1	0	52	318.273	6	.000
	30–39 years	42	35	1	78			
	40–49 years	64	70	0	134			
	> 50 years	2	0	34	36			
Total		159	106	35	300			

The majority of medical practitioners agreed or strongly agreed that medical apps are easy to obtain (mean Likert score = 4.8 ± 0.4); they are looking to obtain more medical apps in the future (4.7 ± 0.5); they would recommend using medical apps to other medical practitioners (4.7 ± 0.5); they do most of their medical learning using medical apps (4.3 ± 1.0); medical apps are essential tools for undergraduate medical students (4.7 ± 0.5); and medical apps are superior to medical textbooks (4.3 ± 0.8). Practitioners also favored medical apps by saying that they are as good as medical books (4.7 ± 0.5); medical apps can replace medical books (4.7 ± 0.5); medical apps supplement medical books (4.6 ± 0.7); medical apps provide useful point-of-care medical information (4.8 ± 0.52); and medical apps use is not dangerous for patient care (4.6 ± 0.6).

### Perceptions of smart device medical apps and its impact on clinical practice

The majority of providers disagreed with or were not sure if medical apps are inferior to medical textbooks (3.1 ± 1.7). The same was true regarding whether free medical apps are inferior in quality to paid apps (3.2 ± 1.7, Table 4).

**Table 4:** Physicians’ perceptions of smart device medical apps

		Frequency	Percent	Mean	SD
Medical apps are easy to obtain	Agree	300	100	4.79	0.408
	Strongly agree				
I am looking to obtain more medical apps in the future	Agree	102	34.0	4.66	0.475
	Strongly agree	198	66.0		
I would recommend the use of medical apps to other medical practitioners	Disagree	3	1.0	4.73	0.506
	Agree	71	23.7		
	Strongly agree	226	75.3		
I do most of my medical learning using medical apps	Disagree	34	11.3	4.32	0.950
	Agree	101	33.7		
	Strongly agree	165	55.0		
Medical apps are essential tools for undergraduate medical studies	Disagree	3	1.0	4.73	0.506
	Agree	71	23.7		
	Strongly agree	226	75.3		
Medical apps are superior to medical textbooks	Not sure	66	22.0	4.33	0.814
	Agree	69	23.0		
	Strongly agree	165	55.0		
Medical apps are as good as medical textbooks	Agree	101	33.7	4.66	0.473
	Strongly agree	199	66.3		
Medical apps are inferior to medical textbooks	Strongly disagree	69	23.0	3.13	1.673
	Disagree	70	23.3		
	Not sure	37	12.3		
	Strongly agree	124	41.3		
Medical apps can replace medical textbooks	Agree	101	33.7	4.66	0.473
	Strongly agree	199	66.3		
Medical apps supplement medical textbooks	Not sure	34	11.3	4.56	0.689
	Agree	64	21.3		
	Strongly agree	202	67.3		
Medical apps provide useful point-of-care medical information	Disagree	4	1.3	4.75	0.518
	Agree	63	21.0		
	Strongly agree	233	77.7		
Free medical apps are inferior in quality to paid apps	Strongly disagree	66	22.0	3.18	1.677
	Disagree	71	23.7		
	Not sure	34	11.3		
	Strongly agree	129	43.0		
There are no dangers in using medical apps for patient care	Disagree	8	2.7	4.60	0.634
	Agree	97	32.3		
	Strongly agree	195	65.0		
	Total	300	100.0		

A majority of medical practitioners agreed or strongly agreed that medical apps improve clinical decision making (4.7 ± 0.6), save time (4.3 ± 1.1), allow for faster access to national clinical practice guidelines (4.3 ± 1.1), allow for faster access to common laboratory reference values (4.7 ± 0.5), help in making differential diagnoses (3.9 ± 1.3), and perform useful medical-related calculations (e.g., estimate creatinine levels; 4.5 ± 0.7). Additionally, medical apps were thought to be beneficial for allowing faster access to reliable medical knowledge sources (4.8 ± 0.4), faster access to reliable clinical skill sources (4.8 ± 0.4), more accurate medication dosing calculations (4.4 ± 1.0), easier medication dosage calculations (4.6 ± 0.7), and faster access to evidence-based medical practices (4.7 ± 0.7, Table 5).

**Table 5:** Perceived impact of smart device medical apps on clinical practice

		Frequency	Percent	Mean	SD
Improve clinical decision-making	Disagree	8	2.7	4.70	0.608
	Agree	65	21.7		
	Strongly agree	227	75.7		
Save time	Disagree	42	14.0	4.26	1.123
	Not sure	34	11.3		
	Agree	29	9.7		
	Strongly agree	195	65.0		
Allow faster access to national clinical practice guidelines	Disagree	41	13.7	4.28	1.106
	Not sure	30	10.0		
	Agree	34	11.3		
	Strongly agree	195	65.0		
Allow faster access to common laboratory reference values	Disagree	5	1.7	4.74	0.543
	Agree	64	21.3		
	Strongly agree	231	77.0		
Help in developing differential diagnoses	Disagree	71	23.7	3.86	1.302
	Not sure	65	21.7		
	Strongly agree	164	54.7		
Perform useful medical related calculations	Not sure	37	12.3	4.52	0.706
	Agree	69	23.0		
	Strongly agree	194	64.7		
Allow faster access to reliable sources of medical knowledge	Agree	71	23.7	4.76	0.426
	Strongly agree	229	76.3		
Allow faster access to reliable sources of clinical skills	Agree	71	23.7	4.76	0.426
	Strongly agree	229	76.3		
Allow accurate calculation of medication dose	Disagree	37	12.3	4.42	0.993
	Agree	64	21.3		
	Strongly agree	199	66.3		
Allow easier calculation of medication dose	Not sure	29	9.7	4.58	0.662
	Agree	68	22.7		
	Strongly agree	203	67.7		
Allow faster access to evidence-based medical practice	Not sure	34	11.3	4.66	0.673
	Agree	34	11.3		
	Strongly agree	232	77.3		
	Total	300	100.0		

### Medical apps commonly used

The medical apps most commonly used by medical practitioners were as follows: Medscape (79%), Oxford medical dictionary (74.3%), Skyscape (69.3%), UpToDate (64.7%), Gray’s Anatomy (63.3%), Epocrates (62.7%), Student BMJ (60.7%), Oxford clinical handbooks (59.3%), Prognosis (57%), and iPharmacy (51.3%). Other medical applications commonly used included Pubmed mobile (48.3%), Differential Diagnosis BMJ (38%), Pocket lab values (30%), ECG guide (30.7%), iStethoscope (29.3%), Micromedex (28%), Eponyms (23%), NEJM (13%), MedCalc (14%), Instant ECG (12%), and Diagnosaurus DDx (12%, Table 6).

**Table 6:** Medical apps used by physicians (*n* = 300)

	Responses	
	n	%
Medscape	237	79.0%
Gray’s Anatomy	190	63.3%
UpToDate	194	64.7%
Pubmed Mobile	145	48.3%
Oxford Medical Dictionary	223	74.3%
Epocrates	188	62.7%
Oxford Clinical Handbooks	178	59.3%
Student BMJ	182	60.7%
Skyscape	208	69.3%
Differential Diagnosis BMJ	114	38.0%
iPharmacy	154	51.3%
Prognosis	171	57.0%
Pocket Lab Values	90	30.0%
ECG Guide	92	30.7%
iStethoscope	88	29.3%
Micromedex	84	28.0%
Eponyms	69	23.0%
NEJM	39	13.0%
Instant ECG	36	12.0%
Diagnosaurus DDx	36	12.0%
MedCalc	42	14.0%

## Discussion

This study examined the use and perceived impact of smart device medical apps in Saudi Arabia. In agreement with studies in other parts of the world [1, 5, 7, 21], we found that medical apps are generally well perceived and are reported by care providers to improve efficiency, accuracy, and education in the clinical setting. The medical apps that are most relied upon in Saudi Arabia (63.3%–79.0% use) were Medscape, Gray’s Anatomy, UpToDate, and Oxford mobile dictionary. These apps were designed to provide medical definitions, review anatomy, and assist clinicians by providing evidence-based answers to clinical questions. This was similar to trends in the United Kingdom, where 86.2% of surgeons reported using medical apps to access online medical resources [22, 23]. These findings indicate that Saudi Arabian physicians believe that medical app use directly improves patient care.

Medical practitioners in Saudi Arabia also agreed or strongly agreed that medical apps are important for medical educations of students, resident physicians, and more experienced practitioners. This is in agreement with several studies that examined the use of medical apps specifically designed for resident training [1, 3, 24–26], continuing medical education [1, 3], and textbook access [1, 3]. Additionally, fast access to medical information was the number one benefit of medical apps reported by students [27], residents [28], and practicing physicians [27]. Resident physicians also commonly use apps to perform medical calculations [28].

The study found that hospitals are also recommending the usage of the medical applications. The finding is similar to Lewis and Wyatt [17, 29] who reported that hospitals are looking for solutions that will aid efficiency both the clinical care and research. The implication of the hospitals recommending use of medical apps is that will increase access to patient records since there are limited desktops at the health care facilities. [30] The study found that there is a significant difference between gender, age, medical rank and frequency of use of installed medical apps. The finding is supported by Rikesh et al., who found that juniors frequently use the medical apps as compared to their seniors and are able to access more information in the medical apps [30]. Medical apps were also accessed by several practitioners for various purposes. The finding is also supported by Rikesh et al. [30], who reported that medical apps are used to access more information related to medicine by health care practitioners.

Our study had several limitations. First, our sample size was relatively small and a study that examines medical app use in a larger group of Saudi Arabian medical practitioners is needed to confirm our findings. Still, our results are in agreement with those found in other regions and provide preliminary information on the benefits of medical apps in Saudi Arabia. Second, our study only examined provider perception of medical apps. Future studies examining regional patient use and perception of medical apps are needed to more completely examine how medical apps impact healthcare. Third, we did not examine differences between urban and rural locations. Because medical apps make care more accessible to patients in rural locations, it would be interesting to examine similarities in and differences between medical app use in urban and rural healthcare locations. Fourth, since temporality of association is a strong criterion for causality, cross-sectional studies cannot prove causality but help to generate causal hypotheses. Lastly, our study did not examine potentially negative impacts of medical app use. Concerns regarding patient safety and confidentiality have been raised [2, 31] and require further investigation.

## Conclusion

In conclusion, the use of medical apps seems to be most beneficial to healthcare practitioners in Saudi Arabia because they make up-to-date medical information more readily available. This information may have a direct impact on patient care, largely because of influences on confirming/assigning diagnoses and determining treatment options. Future studies are needed to better quantify the impact of medical app use on patient care (e.g., correct diagnosis rates, time from diagnosis to treatment, and patient care costs). Studies that examine the impact of medical apps on the patient side in Saudi Arabia are also needed.

## Additional file

Additional file 1: Study Questionnaire. (DOCX 21 kb)
