# Supplementary material for: Popularity and impact of using smart devices in medicine: experiences in Saudi Arabia
Source: BMC Public Health. 2018 Apr 20;18:531. doi: 10.1186/s12889-018-5465-y (PMC5910597; doi:10.1186/s12889-018-5465-y)
Supplement: Supplementary file 1 — Study Questionnaire. (DOCX 21 kb) [file 12889_2018_5465_MOESM1_ESM.docx]

Questionnaire

1. **Data of Participants and purpose to use medical applications:**
2. What is your Gender?

- Male
- Female

1. What is your age (years) ? ……………………………
2. What is your medical rank?

- Resident
- Registrar
- Senior registrar
- Consultant

1. Are you using Smart Devices?

- Yes
- No

1. Are you aware about availability of Med Apps on smart devices

- Yes
- No

1. Have you installed Medical Apps on your smart device?

- Yes
- No

1. Have your hospital/institute ever recommended that you obtain a specific Medical App for your smart device?

- Yes
- No

1. Frequency of use of installed Medical Apps:

- At least once a day
- At least once a week
- At least once a month
- Less than once a month

1. **What is the Purpose of use of installed Medical Apps in your smart device?**

| **Statement** | **Yes** | **No** |
| --- | --- | --- |
| Review of Medical Knowledge |  |  |
| Preparation of Presentations |  |  |
| Look up medical information |  |  |
| Medical journals |  |  |
| During ward rounds |  |  |
| Medical news |  |  |
| Medications or drugs guide |  |  |
| Exam preparation |  |  |
| General clinical textbook |  |  |
| Clinical skills guide |  |  |
| I do not have Medical Apps |  |  |

1. **What is your perceptions about Medical Apps on smart device?**

| **Statement** | **Strongly Agree** | **Agree** | **Not Sure** | **Disagree** | **Strongly Disagree** |
| --- | --- | --- | --- | --- | --- |
| Medical Apps are easy to obtain |  |  |  |  |  |
| I am looking to obtain more Medical Apps in the future |  |  |  |  |  |
| I would recommend the use of Medical Apps to other medical practitioners |  |  |  |  |  |
| I do most of my medical learning using Medical Apps |  |  |  |  |  |
| Medical Apps are essential tools for undergraduate medical studies |  |  |  |  |  |
| Medical Apps are superior to medical books |  |  |  |  |  |
| Medical Apps are as good as medical books |  |  |  |  |  |
| Medical Apps are inferior to medical books |  |  |  |  |  |
| Medical Apps can replace medical books |  |  |  |  |  |
| Medical Apps supplement medical books |  |  |  |  |  |
| Medical Apps provide useful medical information at ‘point-of-care’. |  |  |  |  |  |
| Free Medical Apps are inferior in quality compared to paid apps |  |  |  |  |  |
| No dangers in using Medical Apps for patient care |  |  |  |  |  |

1. **What is your perceptions about impact of Medical Apps on clinical practice?**

| **Statement** | **Strongly Agree** | **Agree** | **Not Sure** | **Disagree** | **Strongly Disagree** |
| --- | --- | --- | --- | --- | --- |
| Improve clinical decision-making |  |  |  |  |  |
| Save time |  |  |  |  |  |
| Allow faster access to national clinical practice guideline |  |  |  |  |  |
| Allow faster access to common laboratory reference values |  |  |  |  |  |
| Help in making differential diagnoses |  |  |  |  |  |
| Perform useful medical related calculations (e.g. estimate creatinine) |  |  |  |  |  |
| Allow faster access to reliable sources of medical knowledge |  |  |  |  |  |
| Allow faster access to reliable sources of clinical skills |  |  |  |  |  |
| Allow accurate medicine dosage calculation |  |  |  |  |  |
| Allow easier medicine dosage calculation |  |  |  |  |  |
| Allow faster access to evidence-based medical practice. |  |  |  |  |  |

1. **What are Medical applications are being used by the (you can select more than one Medical App(**

- Medscape
- Gray’s Anatomy
- Uptodate
- PubMed Mobile
- Oxford Medical Dictionary
- Epocrates
- Oxford Clinical Handbooks
- Student BMJ
- Skyscape
- Differential Diagnosis BMJ
- iPharmacy
- Prognosis
- Pocket Lab Values
- ECG Guide
- iStethoscope
- Micromedex
- Eponyms
- NEJM
- Instant ECG
- Diagnosaurus DDx
- MedCalc
